# Adsorption of Chromium (VI) Using an Activated Carbon Derived from Petroleum Coke Feedstock

**DOI:** 10.3390/ijms232416172

**Published:** 2022-12-18

**Authors:** Kyle S. Fisher, Andrew J. Vreugdenhil

**Affiliations:** 1 Materials Science Graduate Program, Trent University, 1600 W Bank Drive, Peterborough, ON K9L 0G2, Canada; 2 Department of Chemistry, Trent University, 1600 W Bank Drive, Peterborough, ON K9L 0G2, Canada

**Keywords:** adsorption, chromium (VI), activated carbon, adsorption mechanism, nitrogenation, functionalization, thermal treatment

## Abstract

This study aims to determine the main adsorption mechanism by which chromium (VI) is adsorbed onto the surface of a petroleum-coke sourced activated carbon, a feedstock not prevalent in current literature. The study also aims to produce an activated carbon adsorbent that is both cost-effective and efficient for the removal of chromium (VI) in neutral waters. The efficacy of thermally-treated petroleum coke-activated carbon and nitrogenated petroleum coke-activated carbon using ammonium chloride is compared to the efficacy of commercially available activated carbon. X-ray photoelectron spectroscopy of the activated carbons was obtained both before and after exposure to chromium (VI) for characterization of the materials and confirmation of chromium adsorption. The thermally-treated and nitrogenated activated carbons showed significant enhancement of chromium (VI) removal compared to the non-treated petroleum coke-activated carbon (22.4 mg/g, 21.9 mg/g, and 17.0 mg/g, respectively). However, there was no significant difference observed between the thermally-treated and nitrogenated materials. This indicates that the nitrogenation of the surface does not improve the adsorption capacity of the activated carbon, but rather the thermal treatment itself. X-ray photoelectron spectroscopy showed a significant increase in the alcohol functional groups on the surface of the activated carbon material as a result of the heat-treatment process; from 16.02 atomic percent in the non-treated activated carbon to 26.3 atomic percent in the thermally-treated activated carbon. The alcohol functional groups present on the surface allow for chromium (VI) to undergo reduction to chromium (III) under a similar mechanism to the well-known Jones Oxidation Reaction where the reduced chromium (III) species are then physisorbed to the surface of the activated carbon. XPS results are consistent with this as the chromium species present on the surface of the adsorbent is primarily Cr(OH)_3_ (85.6% in the standard AC and 82.5% in the thermally-treated AC). Pseudo-first-order and pseudo-second-order kinetic modeling of the adsorbents indicate that they follow a pseudo-second-order reaction where the rate-limiting step is the chemical sorption of the adsorbate itself.

## 1. Introduction

Representing one of the largest oil deposits in the world, in 2020 the Alberta oil sands in Canada produced approximately 2.98 million barrels, where 1 barrel is approximately 159 L, of crude bitumen per day [1]. The Government of Canada estimates that reserves of 166.3 billion barrels of bitumen are held in the Alberta oil sands; accounting for approximately 10% of the world’s proven oil reserves [2]. Vast quantities of water are required for the bitumen extraction process; a single barrel of oil requires 19.7 barrels of water for the extraction and refining process: 16.4 barrels of recycled water and 3.3 barrels of new water. As the industrial operation is adjacent to the Athabasca River, water is imported from the river for the refining process [3]. As a result of the extraction of bitumen, the resulting processed water becomes alkaline and contaminated with organic acids and heavy metals. Oil sand companies store processed wastewater on-site in tailings ponds that exist at a pH of approximately 8.0. As of 2017, the Government of Alberta reported that tailings ponds cover an area of 220 square kilometers [4]. Due to the large amounts of water used in the process and the resulting contamination, concerns over these tailings ponds have increased over the years. Heavy metal contamination is particularly prominent with metals such as aluminum, arsenic, chromium, nickel, and vanadium [5]. The Government of Canada has documented that the average concentration of *total* chromium in uncontaminated adjacent surface waters is generally below 1 ug/L [6]. The Alberta oil sands report chromium (VI) concentrations in the tailings ponds water reaching up to 2 mg/L [3]. As chromium (VI) is more toxic than chromium (III) and as tailing ponds waters are contaminated by chromium (VI) species rather than chromium (III) species, this study focuses only on the removal of chromium (VI) species as the more acute need. 

Chromium (VI) is highly toxic to both plant and animal life. It is also a widely recognized carcinogen [7]. Toxic effects of chromium show reduced growth in plants, smaller root systems, damage to cell membranes, a decrease or inhibition of seed germination, delayed growth, wilting, and death. In the human body, the excretion of Cr(VI) is very slow due to accumulation in the tissues. This results in the observation of elevated concentrations even decades after exposure has stopped. Toxic effects on humans and animals include developmental issues, promotion of carcinogenesis, as well as damage to the skin, respiratory, reproductive, and digestive systems [8].

Although there are many methods for the removal of metal ions from different matrices; such as ion exchange, coagulation, flocculation, nanofiltration, and chemical precipitation; adsorption has become the preferred method due to its simplicity, cost-effectiveness, and versatility [9,10]. A 2006 review article by Mohan and Pittman [11] provides an extensive review of various adsorbents used for Cr (III) and Cr (VI) adsorption. A 2018 study by Yang et al. [12] investigates the use of cassava sludge sourced activated carbon; a 2020 study by Wang et al. [13] investigates the use of granular activated carbon for mechanisms and modeling of Cr (VI) adsorption, and a 2022 article by Alvarez-Galvan et al. [14] investigates Cr (VI) adsorption using seaweed-sourced activated carbon. Activated carbon sourced from many different feedstocks is investigated in the literature; however, to our knowledge, petroleum coke-sourced activated carbon has only been mentioned as an alternative feedstock proposed for the preparation of activated carbons in Mohan and Pittman’s review article. This research focuses on petroleum coke-sourced activated carbon as a potentially viable, cost-effective, and efficient feedstock, rather than just an alternative feedstock.

Petroleum coke, also known as petcoke, is a waste by-product of the bitumen extraction process from oil sands ore. As of 2011, the processing of oil sands in Alberta produced nearly 10 million tons of petcoke per year. The coal industry and other industries have a total consumption of nearly 5 million tons of petcoke per year. The stockpile of petcoke at the end of the year in 2011 in Alberta was approximately 72 million metric tons of petcoke. Since approximately 15 to 30 percent of a barrel of tar sands bitumen can end up as petcoke [15], this creates an opportunity to use the petcoke produced as a feedstock for a more valuable product by way of efficient activation and effective chemical or thermal tailoring of the surface chemistry to generate economically viable and environmentally valuable activated carbon for environmental remediation.

In this work, petcoke was used to produce an activated carbon using chemical activation with KOH as the activating agent. While the KOH from activation has been shown to be recoverable [16], for this research we have investigated intentionally minimal amounts of KOH to reduce overall cost and environmental impact to generate a commercially viable and highly effective adsorbent from oil sands processing waste materials. All activated carbons used in this study were produced using a 1:1 KOH to petcoke (*w*/*w*) ratio. In the literature, the nitrogenation of an adsorbent’s surface has been shown to enhance the adsorption of chromium (VI) [17,18]. As such, we have investigated the role that nitrogen functionalization of low KOH petroleum coke-sourced activated carbon has on chromium (VI) adsorption. This research provides an avenue for valuable use of waste petcoke and a means of reducing chromium (VI) contamination in mining process water using a cost-effective and efficient material.

Chromium (VI) adsorption was evaluated using this array of petcoke-sourced, low chemical input activated carbons to determine the optimal formulation conditions for Cr (VI) adsorption and to demonstrate in the literature the likely mechanism by which chromium (VI) is adsorbed onto the surface of a given material. A commercially available activated carbon was also used for adsorption and compared as an external benchmark. Evaluation of the remediation materials focused on post-treatment methods of the activated carbons, long-term testing of the efficacy of the activated carbon for chromium (VI) removal, and kinetics evaluation of each activated carbon.

## 2. Results and Discussion

### 2.1. Characterization of AC

The surface composition of the activated carbon and its modification was identified using XPS and is summarized in Table 1. Long-term testing of the activated carbons proved that simply adding more nitrogen to the surface does not increase adsorption. This is shown for example in the NH_4_^+^-Dry AC that needed 29 days to adsorb ~90% Cr (VI) and had a nitrogen content of 3 atom percent compared to the NH_4_^+^-Absorbed AC and Thermally-Treated AC that had a nitrogen content of less than 1 atom percent and adsorbed ~90% Cr (VI) in 16 and 20 days, respectively.

The Cr (VI) adsorption is more likely driven by oxygen speciation on the AC surface. The deconvolution, species assignments, along with the percent of total peak area of the O1s XPS peak signal for each AC sample are presented in Table 2. 

**Figure 1:**
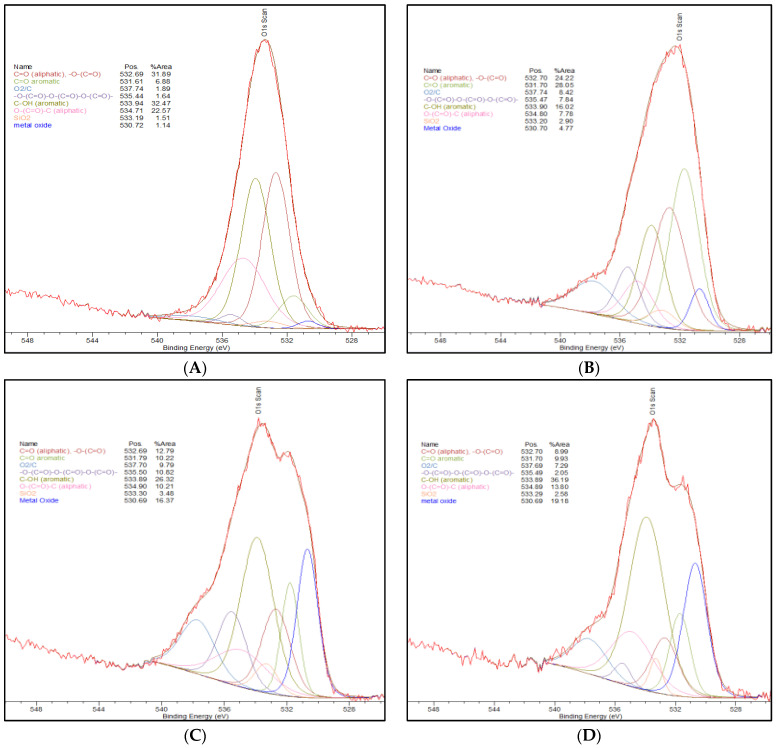

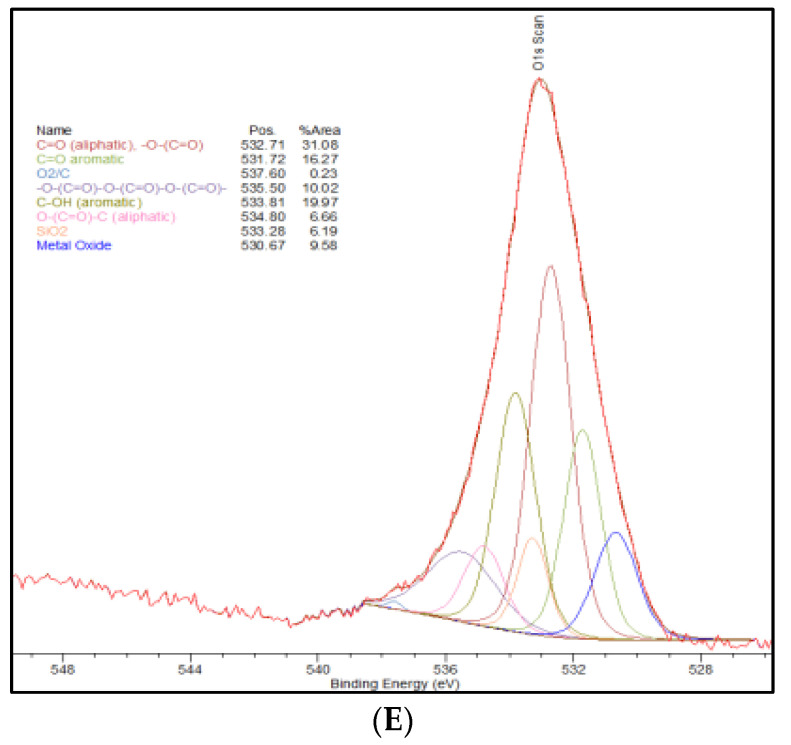
**(A**) O1s XPS peak splitting of Commercial AC. (**B**) O1s XPS peak splitting of Standard AC. (**C**) O1s XPS peak splitting of Thermally-Treated AC. (**D**) O1s XPS peak splitting of NH_4_^+^-Adsorbed AC. (**E**) O1s XPS peak splitting of NH_4_^+^-Dry AC [19,20,21].

In particular, similarities in the C-OH aromatic can be seen for the Commercial AC, NH_4_^+^-Adsorbed AC, and Thermally-Treated AC. The amounts of C-OH aromatic species are increased in these activated carbons compared to the Standard AC. Figure 1, below, shows to XPS spectra of the O1s peak deconvolution for the activated carbon materials that are summarized in Table 2, above.

The C-OH functionality can provide a binding site for the oxophilic chromium. As there is a lower percentage of this oxygen species in the Standard AC, it is expected that the adsorption capacity in the Standard AC is lower. Similarly, for the NH_4_^+^-Dry AC, this species has more C-OH than what is observed in the Standard AC, but which is significantly lower than that observed for the other activated carbon materials. The importance of this speciation is consistent with the fact that the long-term testing results show that NH_4_^+^-Dry AC is more effective with respect to Cr (VI) adsorption than the Standard AC but less effective than the other activated carbons.

Long-term testing of the activated carbons showed no correlation between the amount of nitrogen loaded on the surface and the time required to achieve 90% adsorption. Long-term testing results, including pore size distributions and surface areas of each activated carbon evaluated, can be found in Table 3, below. Based on the long-term testing results, the two functionalized petroleum coke-activated carbons that were the most effective for chromium adsorption were the NH_4_^+^-Adsorbed AC, and the Thermally-Treated AC. As such, these two activated carbons were used in the solution phase adsorption kinetics experiments, along with the Standard AC and the Commercial AC comparators.

### 2.2. Chromium (VI) Kinetics 

**Figure 2:**
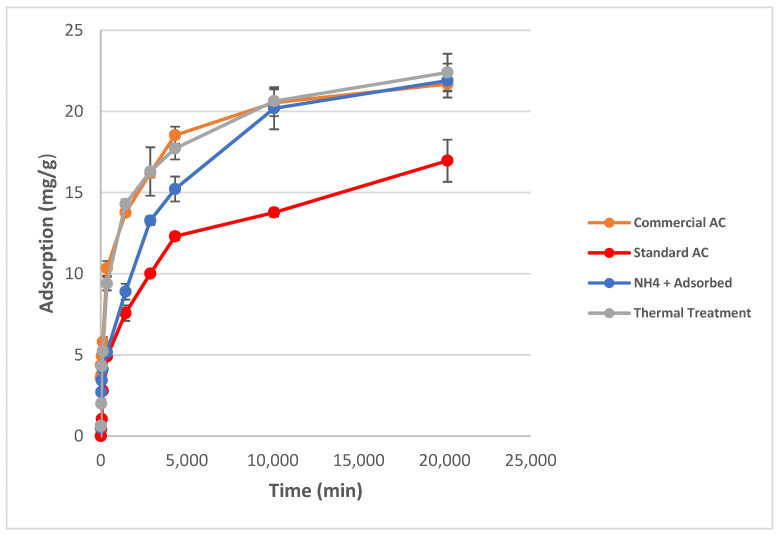
Kinetics curve of the produced activated carbons compared to the commercial AC. Results show 92% adsorption of total chromium (approximately 22 mg/g) in all activated carbons after two weeks with the exception of the standard AC which shows 64% total chromium adsorption (approximately 17 mg/g). Maximum possible adsorption (i.e., 100%) is 25 ± 1 mg/g.

The adsorption kinetics of chromium (VI) using the four different activated carbon materials chosen from the long-term testing was evaluated. Adsorption kinetics over a 2-week time period was determined for the Thermally-Treated AC, NH_4_^+^-Adsorbed AC, Standard AC, and Commercial AC. Chromium (VI) where adsorption equilibrium for each activated carbon system was achieved within the 2-week time period. The final adsorption capacities of the Thermally-Treated, NH_4_^+^-Adsorbed and Commercial ACs were all approximately 92% of the total chromium in solution while the Standard AC was only able to achieve 64% total chromium adsorption after the 2-week period. This can be observed in Figure 2 which shows the kinetics for each of the activated carbon materials and provides the adsorption capacities in mg/g. The kinetics are consistent with the previous observation that the post-activation treatment does generate an increase in the adsorption capacity of the treated activated carbon materials. As described in Section 3.1, this is likely due to the changes in the oxygen functional groups on the surface as a result of the thermal treatment. This also indicates that, since there is no significant change in the adsorption capacities between the Thermally-Treated and the NH_4_^+^-Adsorbed ACs, a simple thermal treatment is just as effective for increasing the adsorption capacity as an ammonium chloride functionalization since the ammonium addition is not causing the increase in the adsorption capacity. Chromium (VI) adsorption kinetics for each of the produced activated carbons indicate that, despite lower surface areas compared to the commercial AC, a petroleum coke-activated carbon with a post-activation thermal treatment matches the adsorption capabilities of the commercial activated carbon on a milligram per gram basis. To remove the dependence of adsorption kinetics on total surface area and isolate the influence of surface functional groups, the adsorption capacity of the activated carbons was also investigated on a milligram of chromium (VI) adsorbed normalized to 1000 m^2^ material surface area. 

**Figure 3:**
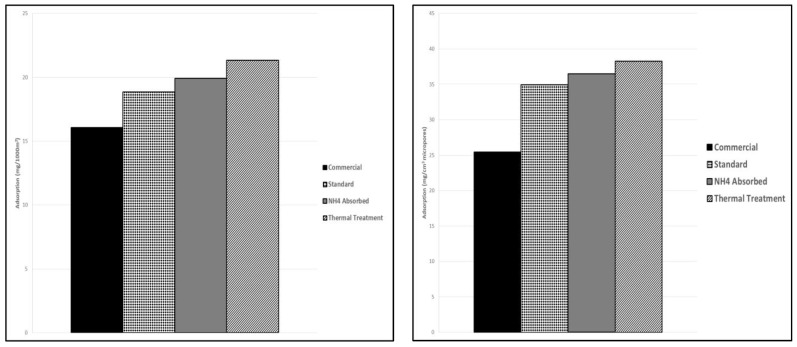
(**Left**) Adsorption capacity of chromium (VI) for activated carbons normalized to 1000 m^2^ surface area. (**Right**) Adsorption capacity of chromium (VI) for activated carbons normalized to 1 cm^3^ pore volume and adjusted for microporosity percentage.

When adsorption results are normalized to 1000 m^2^, the petroleum coke-based activated carbons that were produced showed higher adsorption capacities than the commercially available activated carbon in units of mg Cr (VI) adsorbed/1000m^2^ surface area. This is shown in Figure 3. This indicates that the surface chemistry of the activated carbons plays a more important role in the adsorption than the surface area of the activated carbon as similar adsorption results can be achieved with activated carbons of lower surface area by simply changing the surface chemistry. 

Normalization to micropore volume was also carried out to investigate if the pore size plays a significant role in adsorption. Microporosity of the commercial activated carbon makes up 56% of the total pore volume while microporosity of the Standard AC, NH_4_^+^-Adsorbed, and Thermally-Treated are 79%, 74%, and 76%, of the total porosity, respectively. The adsorption capacities of the activated carbons in terms of microporosity relative to 1 cm^3^ total pore volume shown in Figure 2 indicates that the more microporous samples have higher adsorption capacities per micropore area. All normalized data can be found in Appendix A, Table A1.

### 2.3. Kinetic Modelling

Pseudo-first and pseudo-second-order kinetic modeling was performed for each of the activated carbons following Langmuir adsorption kinetics using the information from the kinetics curve found in Figure 1. A review article by Revellame et al. [22] shows how pseudo-first-order kinetic modeling should be linear with respect to the equation:

(1) $$ln\left[q_{e}-q\left(t\right)\right]=-k_{1}t+lnq_{e}$$

where *q(t)* and *q_e_* are the amount (in mg/g) of adsorbate adsorbed at any given time, *t*, and at equilibrium, respectively, and where *k*_1_ is the rate constant for the pseudo-first-order kinetic model. Pseudo-second-order kinetic modeling was shown to be linear with respect to the equation:

(2) $$\frac{t}{q\left(t\right)}=\left(\frac{1}{q_{e}}\right)t+\frac{1}{k_{2}q_{e}^{2}}$$

where *q(t)* and *q_e_* are the amount (in mg/g) of adsorbate adsorbed at any given time, *t*, and at equilibrium, respectively, and where *k*_2_ is the rate constant for the pseudo-second-order kinetic model. Table 4 presents the summary of the kinetic modeling for each of the activated carbons. 

If the activated carbon adsorption kinetics follows a pseudo-first-order model, it indicates that the adsorption behaves as a first-order reaction where one reactant is present in excess. If the activated carbon adsorption kinetics follows a pseudo-second-order model, it indicates that the rate-limiting step is the chemical adsorption itself, in other words, the adsorption rate is dependent on the adsorption capacity of the adsorbent not on the concentration of the adsorbate. 

For the commercial activated carbon, it can be found that the fit is better with the pseudo-second-order modeling compared to the pseudo-first-order modeling. This can clearly be seen as the linear relationship is higher in the second-order model. This is also consistent with the *q_e_* value that is calculated using this model. The *q_e_* value obtained using this model was 21.79 mg/g while the kinetics curve for the commercial AC showed equilibrium adsorption after 2 weeks to be 21.69 mg/g. It is also clear by the adsorption rate constant, *k*, that the commercial AC follows the pseudo-second-order kinetic model because the adsorption rate constant *k*_2_ is lower than *k*_1_ from the pseudo-first-order model. 

When observing the standard activated carbon, it also fits well with the pseudo-second-order modeling with a linear relationship greater in the pseudo-second-order model than in the pseudo-first-order model. The calculated *q_e_* for the pseudo-second-order model, 17.45 mg/g, is also similar to the experimental equilibrium obtained from the kinetics curve after 2 weeks of 16.96 mg/g. Finally, the rate constant *k*_2_ was also calculated to be lower than the rate constant *k*_1_, indicating that the rate is limited to the pseudo-second-order model. 

The NH_4_^+^-Adsorbed activated carbon shows better linearity for the pseudo-first-order model; however, both the *q_e_* and *k* values for the pseudo-second-order model show that the second-order model is a better fit. The *q_e_* value calculated under the pseudo-second-order model is much closer to that which was determined in the kinetics experiments, and the *k*_2_ adsorption rate constant is lower than the *k*_1_ adsorption rate constant, indicating that the adsorption rate is dependent on the chemical sorption itself, rather than the excess concentration of a reactant.

**Figure 4:**
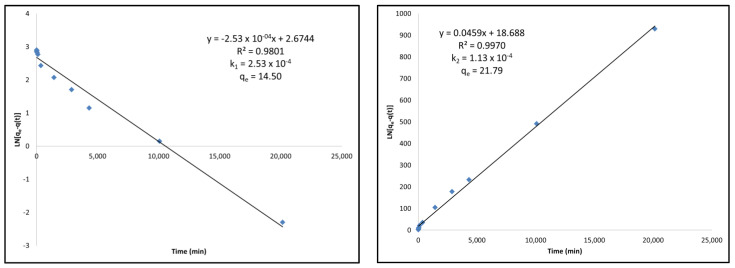
(**Left**) Pseudo-first-order kinetic modeling of commercial activated carbon under Langmuir adsorption kinetics equations. (**Right**) Pseudo-second-order kinetic modeling of commercial activated carbon under Langmuir adsorption kinetics equations.

**Figure A1:**
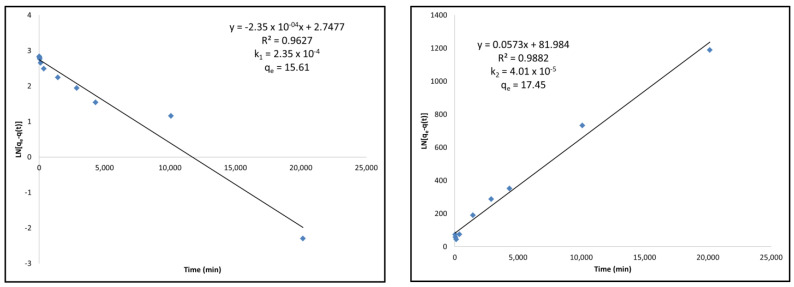
(**Left**) Pseudo-first-order kinetic modeling of standard activated carbon under Langmuir adsorption kinetics equations. (**Right**) Pseudo-second-order kinetic modeling of standard activated carbon under Langmuir adsorption kinetics equations.

**Figure A2:**
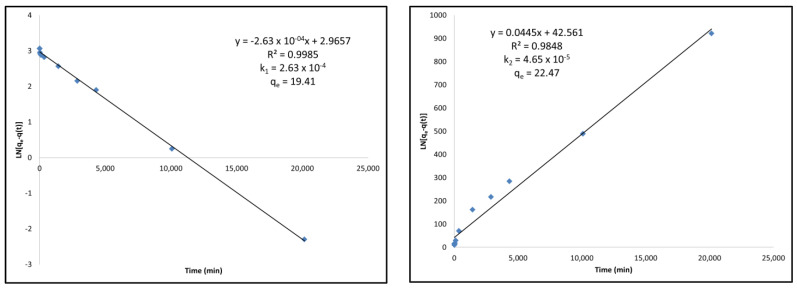
(**Left**) Pseudo-first-order kinetic modeling of NH_4_^+^-Adsorbed activated carbon under Langmuir adsorption kinetics equations. (**Right**) Pseudo-second-order kinetic modeling of NH_4_^+^-Adsorbed activated carbon under Langmuir adsorption kinetics equations.

**Figure A3:**
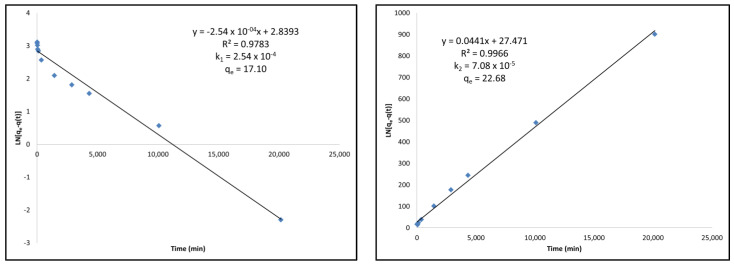
(**Left**) Pseudo-first-order kinetic modeling of thermally-treated activated carbon under Langmuir adsorption kinetics equations. (**Right**) Pseudo-second-order kinetic modeling of thermally-treated activated carbon under Langmuir adsorption kinetics equations.

Finally, the thermally-treated activated carbon follows a pseudo-second-order model more accurately than the pseudo-first-order model. The linearity in the pseudo-second-order model is much greater, the *q_e_* value calculated from the model is much more accurate than that which was determined in the kinetics experiment, and the *k*_2_ adsorption rate constant is lower than the *k*_1_ determined from the pseudo-first-order model. Figure 4 shows an example of the pseudo-first-order and pseudo-second-order modeling for commercial activated carbon. All other kinetic modelings can be found in Appendix B, Figure A1, Figure A2 and Figure A3.

### 2.4. Proposed Adsorption Mechanism

**Figure 5:**
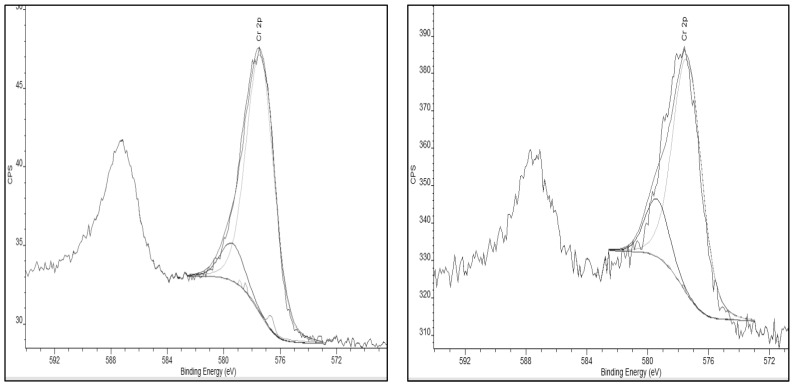
(**Left**) Cr2p peak of Standard Activated Carbon, post-chromium adsorption. (**Right**) Cr2p peak of the Thermally-Treated Activated Carbon post-chromium adsorption.

XPS spectra were obtained of the chromium species present on the activated carbon materials following exposure to chromium (VI) containing aqueous solutions. The results for the Cr2p spectral region are presented in Figure 5. XPS spectra of the activated carbon before exposure to chromium showed no peak in the 572 to 584 eV binding energy region. This confirms that chromium adsorption is occurring as a peak in this region is present after the activated carbons’ exposure to chromium. The deconvolution, species assignments, and the percent of total peak area of the Cr2p XPS signal for each chromium species observed on the standard AC and the Thermally-Treated AC samples are presented in Table 5.

As shown in Table 5, the chromium species on the AC substrates are predominantly chromium hydroxide (Cr(OH)_3_) for both surfaces. Note, that the Cr_2_O_3_ species identification is made more complex by the well-known XPS multiplet splitting for this and similar species as described by Biesinger, et al. [24] in their XPS study of chromium compounds. Most importantly, the chromium VI species to which the AC surface was exposed are extensively converted to chromium III following adsorption. In addition, chromium adsorption is dependent on the surface functionality of the carbon. The thermal treatment of activated carbon substrate results in an increase in the number of C-OH functional groups on the carbon surface as shown in Table 6. Thermal treatment of the activated carbon substrate results in a substantial increase in the C-OH functionality from 16% to 26% of the O1s peak area. This increase, in turn, results in an increase in the adsorption of chromium species onto the surface as presented in the kinetics of adsorption displayed previously in Figure 1 with a maximum total chromium adsorption of 64% for the standard AC and 93% for the thermally treated AC.

**Figure 6:**
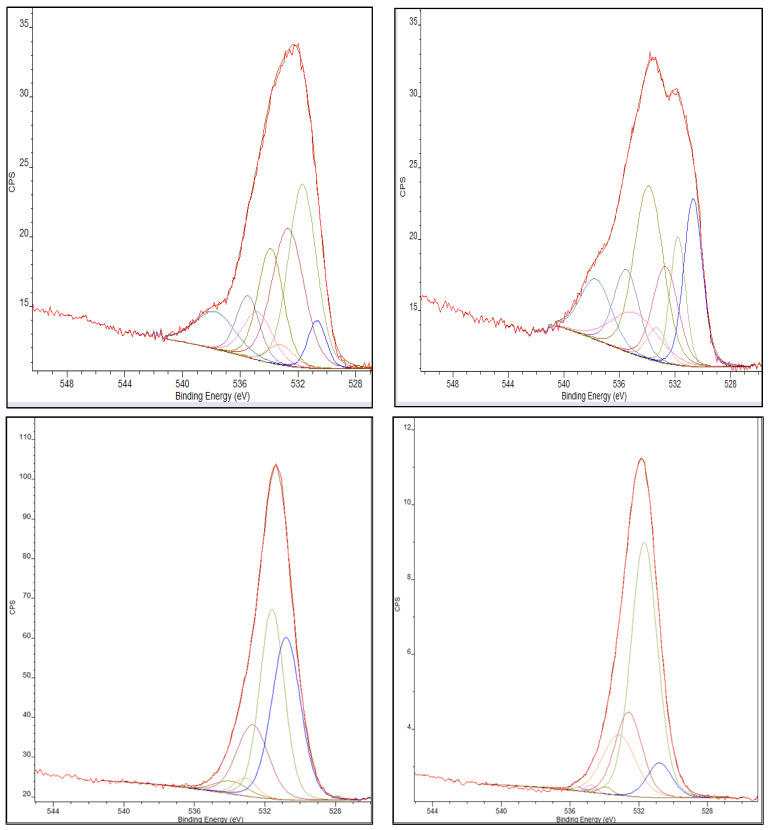
(**Top Left**) O1s deconvolution of standard AC pre-exposure to chromium (VI). (**Bottom Left**) O1s deconvolution of standard AC post-exposure to chromium (VI). (**Top Right**) O1s deconvolution of thermally-treated AC pre-exposure to chromium (VI). (**Bottom Right**) O1s deconvolution of thermally-treated AC post-exposure to chromium (VI).

Upon chromium adsorption, a substantial decrease in the C-OH functionality is observed from 16% to 3% and 26% to less than 1% for the standard and thermally treated AC, respectively. An increase in carbonyl functionality is also detected for the standard and thermally treated AC species following chromium exposure. Figure 6 shows the O1s peak deconvolution of the standard AC and the thermally-treated AC both before and after exposure to Cr (VI).

**Figure 7:**
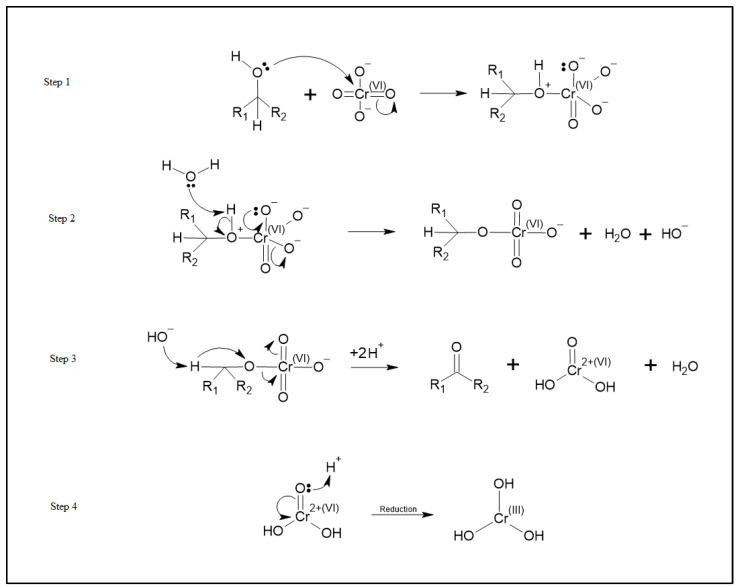
The adsorption schematic demonstrating the reduction of chromium (VI) in solution to chromium (III) hydroxide and the oxidation of alcohols to ketones on the activated carbon surface.

Based on these observed changes in oxidation states and the effect of thermal treatment of the carbon, we propose a redox adsorption process as shown in Figure 7. This process is similar to the known organic Jones Oxidation reaction mechanism for the reaction of HCrO_4_^−^ with an alcohol where HCrO_4_^−^ reacts with an alcohol to form a ketone and an unstable chromium VI species, CrO(OH)_2_. Further reactions then reduce this chromium (VI) species to Cr (III) [25]. 

Speciation of chromium (VI) at pH 8.0 is predominantly chromate ions (CrO_4_^2−^) [26]. As such, we propose that chromate ions (CrO_4_^2−^) react with the OH species on the surface of the activated carbon materials to form ketones on the carbon surface and the unstable CrO(OH)_2_ species. This unstable species is then reduced to Cr(OH)_3_ which is adsorbed onto the activated carbon materials. This mechanism shows how an increase in the alcohol functional groups on the surface can increase the adsorption of chromium by providing more sites at which this mechanism can occur. The proposed adsorption mechanism for chromium adsorption is shown in Figure 7.

**Figure 8:**
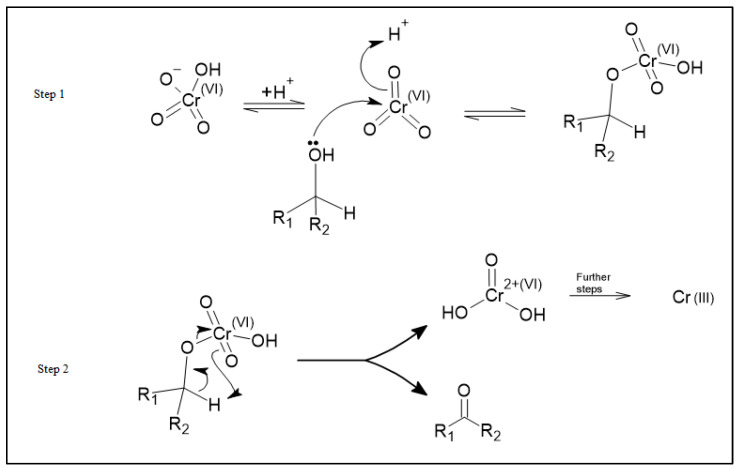
The Jones Oxidation reaction mechanism sourced from Organic Chemistry 2nd Edition by Clayden et al. Mechanism found on page 544.

As previously mentioned, the proposed reaction above is very similar to the well-known Jones Oxidation reaction. The Jones Oxidation reaction mechanism is shown in Figure 8. When comparing these two mechanisms, steps 1 and 2 found in Figure 7 are very similar to step 1 found in Figure 8. The resulting product of these steps is a chromate ester. Similarly, step 3 found in Figure 7 is very similar to step 2 of Figure 8 where the result of the reaction is a ketone and the chromium (VI) species, CrO(OH)_2_. Finally, step 4 of Figure 7 is consistent with the “further steps” portion of the Jones Oxidation reaction found in Figure 8 where Cr (VI) is reduced to Cr (III). It is important to note that the Jones Oxidation reaction is an organic mechanism that focuses on the formation of ketone from alcohol. While the transformation of alcohol to a ketone is important, this study is more focused on the reduction of chromium (VI) to chromium (III).

The net result of this process is the reduction of the chromium (VI) species to chromium (III) and the conversion of the OH to a carbonyl species on the surface. In our proposed mechanism, the Cr(OH)_3_ species is then physisorbed onto the activated carbon surface through hydrogen bonding between the hydrogen atoms on the Cr(OH)_3_ species and the electron-rich oxygen centers of the carbonyl functional groups of the activated carbon. 

## 3. Materials and Methods

The feedstock petroleum coke was obtained from Suncor, Canada, and compared to a commercial activated carbon obtained from Strem Chemicals. Laboratory grade, 99.5% purity, chromium (VI) oxide was purchased from Acros Organics, Morris Plains, NJ, USA, and was used for all chromium solutions. Reagent grade, 99.5% purity, ammonium chloride was purchased from Saint-Léonard, QC, Canada, and was used for all functionalizations requiring ammonium chloride doping. 

### 3.1. Activation of Petroleum Coke

Petroleum coke (PC) was ground to pass a 0.58 mm mesh and underwent preliminary heating in air at 400 °C for 1 h. The heated PC was mixed with solid KOH at a 1:1 ratio (*w*/*w*) and heated under flowing nitrogen from room temperature to 900 °C over 8 min and held for 5 min at 900 °C. The resulting activated carbon was ground with a mortar and pestle and washed with 10 mL of deionized water for every 1 g of initial feedstock at 80 °C for 1 h with stirring at 200 rpm. The product was vacuum-filtered and rinsed with 10 mL of 0.1 M HCl for every 1 g of initial feedstock and dried at 110 °C overnight.

### 3.2. Post-Treatments of the Activated Carbon

Several different post-activation functionalizations were carried out as follows. Functionalization of the activated carbon was carried out using water reflux, brominated activated carbon reflux, dry mixing, and solution adsorption. 

#### 3.2.1. Ammonium Chloride Water Refluxed AC (NH_4_^+^-H_2_O-Reflux)

Fifteen grams of AC was placed in a round bottom flask with 300 mL of “constant boiling” hydrobromic acid (aqueous solution containing 47.6% HBr by mass) and refluxed for 1 h before being filtered and washed with acetonitrile. The resulting brominated activated carbon was then mixed with dry NH_4_Cl and deionized water in a 1:1:10 by mass ratio, respectively, and refluxed for 1 h. The resulting solids were filtered and dried at 110 °C overnight. 

#### 3.2.2. Ammonium Chloride Dichloromethane Reflux (NH_4_^+^-CH_2_Cl_2_-Reflux)

Fifteen grams of AC was placed in a round bottom flask with 300 mL of “constant boiling” hydrobromic acid and refluxed for 1 h before being filtered and washed with acetonitrile. The resulting brominated activated carbon was then mixed with dry NH_4_Cl and dichloromethane in a 1:1:10 by mass ratio, respectively, and refluxed for 1 h. The resulting solids were filtered and dried at 110 °C overnight. 

#### 3.2.3. Ammonium Chloride Dry Mixture AC (NH_4_^+^-Dry)

Petroleum coke-activated carbon was mixed with dry ammonium chloride in a 1:1 mass ratio and thermally treated under flowing nitrogen at 550 °C for 30 min. 

#### 3.2.4. Ammonium Chloride Adsorbed AC (NH_4_^+^-Adsorbed) 

Petroleum coke-activated carbon was mixed with dry NH_4_Cl and deionized water at a 1:1:2 by mass ratio. The resulting slurry was allowed to stir for 24 h before being vacuum filtered and dried at 110 °C. Following drying, samples were thermally treated under flowing nitrogen at 550 °C for 30 min. 

#### 3.2.5. Thermal Treatment (Thermally-Treated)

Petroleum coke-activated carbon was mixed with deionized water in a 1:2 by mass ratio and allowed to stir for 24 h before being vacuum filtered and dried at 110 °C. The samples were then thermally treated under flowing nitrogen at 550 °C so that a comparison of a thermal-treatment with, and without the addition of ammonium could be compared. 

### 3.3. Microwave Plasma Atomic Emission Spectrometry Analysis (MP-AES) 

Chromium (VI) calibration standards of 1, 2, 5, 10, 25, and 75 ppm were prepared in 0.1 M tris(hydroxymethyl)aminomethane (more commonly referred to as tris base) buffer to construct calibration curves used during MP-AES analysis. Calibration curves were created each new time the instrument was used under acceptable tolerance of 10% calibration error and linearity (R^2^) above 0.9900. All standards and samples were analyzed in triplicate with a sample uptake time of 30 s with a delivery speed of 80 rpm followed by a stabilization time of 15 s with a delivery speed of 15 rpm. Readings were integrated over a 10 s period and chromium emission was detected at a wavelength of 425.433 nm [27]. MP-AES operating conditions are given in Table 7. 

### 3.4. Chromium (VI) Study 

A 1000 ppm stock solution of chromium (VI) in deionized water was prepared from chromium (VI) oxide. All samples and standards were prepared by serial dilution from the 1000 ppm stock solution. All samples were prepared to a concentration of 50 ppm chromium (VI) with a final volume of 50 mL using 0.1 M tris base buffer to dilute the samples. Tris base buffer was used to ensure that the pH of the chromium solution did not shift after the addition of the activated carbon, but stayed between 7.0 and 8.0. 

#### 3.4.1. Long-Term Testing

A comparison of the efficiency of each activated carbon was conducted through long-term adsorption testing at a solution pH of 8. To evaluate the efficiency of the activated carbon to remove chromium from the solution, samples of 50 ppm chromium (VI) were prepared in 0.1 M tris base buffer to a total volume of 50 mL and 0.1 g of activated carbon was added to the samples. Samples were allowed to sit until the solution color was nearly colorless (i.e., a slight tinge of yellow remaining). Once solutions became nearly colorless, the sample was filtered using a 0.45 μm filter and analyzed using MP-AES for chromium quantitation. 

#### 3.4.2. Chromium (VI) Kinetics 

Adsorption kinetics of the chromium (VI) were obtained using the 30 min 550 °C thermally-treated petroleum coke activated carbon, the 30 min 550 °C ammonium chloride adsorbed activated carbon and the commercial activated carbon. Times of 5 min, 10 min, 30 min, 1 h, 2 h, 6 h, 24 h, 48 h, 72 h, 1 week, and 2 weeks were tested; 0.1 g of activated carbon was added for adsorption and all samples were allowed to stir for their respective time at a stirring speed of 150 rpm before being filtered using a 0.45 μm filter and analyzed on the MP-AES for chromium quantitation. 

### 3.5. Surface Texture Characterization of AC 

#### 3.5.1. Nitrogen Adsorption Measurements 

Measurement of the surface area was carried out using gas phase nitrogen adsorption measurements on a Tristar II plus (Micromeretics, Ottawa, Ontario, Canada). The samples were analyzed using N_2_ adsorption at 77 K with 50 points monitoring adsorption between 0.0065 p/p^0^ and 0.995 p/p^0^ and 52 points desorption between 0.995 p/p^0^ and 0.104 p/p^0^. Some samples were additionally investigated using CO_2_ adsorption at 273 K. All surface areas are reported using Brunauer–Emmett–Teller surface area analysis with pore size distributions developed using DFT with slit geometry modeling 2D-NLDFT with N2 carbon finite pores. 

#### 3.5.2. X-ray Photoelectron Spectroscopy (XPS) 

Measurement of XPS spectra was made on an AXIS Supra spectrometer (Kratos, Spring Valley, New York, NY, USA). A monochromatic AlKα source (15 mA, 15 kV) was used with the instrument work function calibrated to give a binding energy (BE) of 83.96 eV for the Au4f_7/2_ line for metallic gold. The spectrometer dispersion was adjusted to give a BE of 932.62 eV for the Cu2p_3/2_ line of metallic copper. The Kratos charge neutralizer system was used on all specimens and survey scan analyses were carried out with an analysis area of 300 × 700 microns with a pass energy of 160 eV. A pass energy of 20 eV with an analysis area of 300 × 700 microns was carried out for high-resolution analyses. Fitting of peaks was conducted using CASA XPS (version 2.31) with the spectra being corrected to the main line of the carbon 1s spectrum at 284.8 eV.

## 4. Conclusions

An efficient, cost-effective activated carbon that can be produced on a large scale was produced for chromium (VI) adsorption using a simple thermal treatment on petroleum coke-derived activated carbon. Thermal-treatment of the activated carbons produced in the lab resulted in a shift in the oxygen speciation that allowed for increased adsorption capabilities, equivalent to that of commercial activated carbon material. Adsorption capacity comparisons normalized to surface area and pore volume basis showed that functionality of the activated carbons and higher microporosity percentages increased the adsorption capacities and that these factors are more beneficial than having a high surface area. Kinetics studies of the activated carbons produced in the lab compared to that of the commercial activated carbon showed varying degrees of chromium (VI) adsorption during the initial kinetics with no significant difference observed at kinetics periods after one week in all but the Standard AC. Kinetic modeling shows that the adsorbents follow a pseudo-second-order kinetic model indicating that the adsorption rate is dependent on the sorption itself [22]. It was also determined that the addition of the ammonium chloride was not the cause of the increased adsorption observed but rather the shift in the oxygen speciation. XPS of the activated carbons shows that the main factor in the increased adsorption is a change in the oxygen speciation as a result of the post-activation thermal treatment. By increasing the alcohol functional groups through the thermal treatment, we provide more sites for which chromate ions can undergo a similar mechanism to the well-known Jones Oxidation mechanism where alcohol reacts with chromium (VI) species to form ketones and reduce the chromium (VI) to chromium (III) [25]. In this case, the increased alcohol functionality on the activated carbon surface allows chromate ions to be reduced to Cr(OH)_3_ where it is subsequently adsorbed to the ketone-rich surface of the activated carbon through physical adsorption, mainly hydrogen bonding. 

## Figures and Tables

**Table 1:** XPS atom percent surface composition of activated carbon.

Sample	Atom % C	Atom % O	Atom % N	Atom % Si	Atom % S	Atom % Br
Standard AC:	77.63	17.04	0.39	2.17	0.42	0.00
NH_4_^+^-H_2_O-Reflux:	88.64	9.30	0.62	0.85	0.60	0.15
NH_4_^+^-CH_2_Cl_2_-Reflux:	86.43	10.29	0.95	1.51	0.56	0.14
NH_4_^+^-Dry:	80.35	11.36	3.12	3.70	0.78	0.00
NH_4_^+^-Adsorbed:	88.86	8.03	1.64	0.84	0.63	0.00
Thermally-Treated AC:	80.60	16.29	0.67	1.87	0.00	0.00
Commercial AC:	85.26	14.74	0.00	0.00	0.00	0.00

**Table 2:** Oxygen atom percent speciation from deconvolution of the XPS O1s peak for the various activated carbons investigated [19,20,21].

Species:	Binding Energy (eV):	Standard AC (Percent Integrated Peak Area):	NH_4_^+^-Dry AC (Percent Integrated Peak Area):	NH_4_^+^-Adsorbed AC (Percent Integrated Peak Area):	Thermally-Treated AC (Percent Integrated Peak Area):	Commercial AC (Percent Integrated Peak Area):
C=O (aliphatic), -O-(C=O)	532.70	24.22	31.08	8.99	12.79	31.89
C=O (aromatic)	531.70	28.05	16.27	9.93	10.22	6.88
O_2_/C	537.75	8.42	0.23	7.29	9.79	1.89
-O-(C=O)-O-(C=O)-O-(C=O)-	535.50	7.84	10.02	2.05	10.82	1.64
C-OH (aromatic)	533.90	16.02	19.97	36.19	26.32	32.47
O-(C=O)-C (aliphatic)	534.80	7.78	6.66	13.80	10.21	22.57
SiO_2_	533.20	2.90	6.19	2.58	3.48	1.51
Metal Oxide	530.70	4.77	9.58	19.18	16.37	1.14

**Table 3:** The number of days required for various activated carbons to effectively remove chromium (VI) from solution while comparing their surface area and pore size distributions.

Activated Carbon	Number of Days to Adsorb 90% Total Cr(VI) in Solution	Surface Area (m^2^/g)	Pore Size Distribution (Total Pore Volume: Micropore Volume) (cm^3^/g:cm^3^/g)
Standard AC	100 ^1^	900	0.383:0.302
NH_4_^+^-H_2_O-Reflux	29	1020	0.467:0.319
NH_4_^+^-CH_2_Cl_2_-Reflux	55	860	0.364:0.276
NH_4_^+^-Dry	29	930	0.406:0.301
NH_4_^+^-Adsorbed	16	1100	0.445:0.330
Thermally-Treated	20	1050	0.444:0.337
Commercial AC	16	1350	0.480:0.270

^1^ After 100 days, 90% adsorption was still not achieved and it was concluded that adsorption had reached equilibrium and would not increase further.

**Table 4:** A summary of the pseudo-first and pseudo-second-order kinetic modeling for each of the activated carbons that underwent kinetics experiments.

Activated Carbon:	Pseudo-First or Pseudo-Second Order Model	Model Equation	Linearity (R^2^) of the Model	k Value	q_e_ value
Commercial AC	First	y = −2.53 × 10^−4^x + 2.6744	0.9801	2.53 × 10^−4^	14.50
	Second	y = 0.0459x + 18.688	0.9970	1.13 × 10^−4^	21.79
Standard AC	First	y = −2.35 × 10^−4^x + 2.7477	0.9627	2.35 × 10^−4^	15.61
	Second	y = 0.0573x + 81.984	0.9882	4.01 × 10^−5^	17.45
NH_4_^+^-Absorbed AC	First	y = −2.63 × 10^−4^x + 2.9657	0.9985	2.63 × 10^−4^	19.41
	Second	y = 0.0445x + 42.561	0.9848	4.65 × 10^−5^	22.47
Thermally-Treated AC	First	y = −2.54 × 10^−4^x + 2.8393	0.9783	2.54 × 10^−4^	17.10
	Second	y = 0.0441x + 27.471	0.9966	7.08 × 10^−5^	22.68

**Table 5:** Chromium atom percent speciation from deconvolution of the XPS Cr2p 3/2 peak post chromium adsorption [19,20,23].

Species	Binding Energy (eV)	Standard AC (Percent Integrated Peak Area)	Thermally-Treated AC (Percent Integrated Peak Area)
Cr_2_O_3_	575.60, 576.60, 577.40, 578.40, 579.20	3.36	0.75
Cr(OH)_3_	577.20	85.63	82.45
Cr (VI) mixed species	579.40	11.00	16.81

**Table 6:** Oxygen atom percent speciation from deconvolution of the XPS O1s peak pre- and post-chromium adsorption [19,20,21].

	C=O (Aliphatic)	C=O (Aromatic)	C-OH (Aromatic)
Standard AC Pre-Cr Adsorption:	24.22	28.05	16.02
Standard AC Post-Cr Adsorption:	18.33	37.28	3.40
Thermally-Treated AC Pre-Cr Adsorption:	12.79	10.22	26.32
Thermally-Treated AC Post-Cr Adsorption:	18.70	55.92	0.75

**Table 7:** MP-AES operating conditions for the analysis of chromium (VI).

Instrument Parameter	Operating Condition
Spray Chamber	Cyclonic single-pass
Sample Uptake (s)	30
Stabilization (s)	15
Read Time (s)	10
Number of Replicates	3
Background Correction	Auto
Wavelength (nm)	425.433
Ignition Gas	Argon
Carrier Gas	Nitrogen
Carrier Gas Flow Rate (L/min)	0.70
Calibration Fit	Linear
Acceptable Linear Tolerance	≥0.9900
Calibration Error	10%

## Appendix A

**Table A1:** Summary of activated carbon adsorption capacities after 2 weeks normalized to grams (mg/g), surface area (mg/1000 m^2^), and microporosity (mg/cm^3^ micropores).

Activated Carbon:	Adsorption Capacity (mg/g):	Surface Area:	Adsorption (mg/1000 m^2^):	Micropore Percentage:	Adsorption (mg/cm^3^ Micropores):
Standard AC	16.96	900	18.85	79%	34.92
NH_4_^+^-Adsorbed AC:	21.90	1100	19.91	74%	36.49
Thermally-Treated AC:	22.39	1050	21.33	76%	38.29
Commercial AC:	21.69	1350	16.07	56%	25.42
